# Planning and Performance in Small Groups: Collective Implementation Intentions Enhance Group Goal Striving

**DOI:** 10.3389/fpsyg.2017.00603

**Published:** 2017-04-19

**Authors:** J. Lukas Thürmer, Frank Wieber, Peter M. Gollwitzer

**Affiliations:** ^1^Department of Politics and Public Administration, University of KonstanzKonstanz, Germany; ^2^Department of Psychology and Learning Research and Development Center, University of Pittsburgh, PittsburghPA, USA; ^3^Department of Psychology, University of KonstanzKonstanz, Germany; ^4^Zurich University of Applied Sciences, School of Health ProfessionsZurich, Switzerland; ^5^Department of Psychology, New York University, New YorkNY, USA

**Keywords:** cooperation and interaction, collective implementation intentions, small group performance, motivation, physical persistence

## Abstract

There are two key motivators to perform well in a group: making a contribution that (a) is crucial for the group (indispensability) and that (b) the other group members recognize (identifiability). We argue that indispensability promotes setting collective (“We”) goals whereas identifiability induces individual (“I”) goals. Although both goals may enhance performance, they should align with different strategies. Whereas pursuing collective goals should involve more cooperation, pursuing individual goals should involve less cooperation. Two experiments support this reasoning and show that planning out collective goals with *collective implementation intentions* (cIIs or “We-plans”) relies on cooperation but planning out individual goals with individual *implementation intentions* (IIs or “I-plans”) does not. In Experiment 1, three-member groups first formed a collective or an individual goal and then performed a first round of a physical persistence task. Groups then either formed a respective implementation intention (cII or II) or a control plan and then performed a second round of the task. Although groups with cIIs and IIs performed better on a physical persistence task than respective control groups, only cII groups interacted more cooperatively during task performance. To confirm the causal role of these interaction processes, Experiment 2 used the same persistence task and manipulated whether groups could communicate: When communication was hindered, groups with cIIs but not groups with IIs performed worse. Communication thus qualifies as a process making cIIs effective. The present research offers a psychology of action account to small group performance.

## Introduction

Imagine lifting a heavy ball together with team mates in a small group. You realize that the other group members could not lift the ball without your help. You feel energized and try really hard because you know that your group needs you and that your contribution really makes a difference. Now imagine the same situation from a different perspective: You are in your group and you realize that the other group members can see your contribution. If you are the first to give up holding the ball, everybody will know that it was you who failed; if you push through until somebody else gives up, everybody will notice that too. Again, you feel energized and try really hard because you want to do better than the other group members. These two perspectives^1^ reflect the two most commonly studied motivators in small groups (Karau and Williams, 1993; Kerr and Hertel, 2011): indispensability (your group needs you) and identifiability (the other group members can recognize your contribution).

Indispensability leads group members to focus on outcomes for the entire group (i.e., we-goals), and accordingly group members use cooperative behaviors to attain these goals. In contrast, identifiability leads group members to focus on outcomes for oneself (i.e., I-goals), and accordingly group members may attain these goals without cooperation. Individuals attain and strive for their goals more successfully when they form additional if-then plans (implementation intentions, IIs). We argue that groups can use such if-then plans in two ways: support indispensability-related we-goals with new we-plans (collective implementation intentions, cIIs) or identifiability-related I-goals with traditional I-plans. Both types of plans should enhance performance, but only we-plans should increase cooperative group interaction.

### Indispensability versus Identifiability: Setting Collective versus Individual Goals

Small group research has identified two primary motivators for group members to perform well: (a) one’s contribution is crucial for the group (*indispensability*) and (b) the other group members can recognize one’s contribution (*identifiability*) (Karau and Williams, 1993; Kerr and Hertel, 2011). Indispensability is motivating because one expects to make a crucial contribution to a valued group outcome or result (Kerr and Hertel, 2011). Such positive outcomes include attaining a group performance goal, receiving a group reward, or winning against another team. Because these outcomes all apply to the entire group, the goal matching this mechanism is best described as collective (e.g., “We want to break the record”). If the group attains the collective goal, all group members benefit and one group member’s contributions benefit all other group members as well. Therefore, collective goals triggered by indispensability have a positive interdependence within the group (Deutsch, 1949).

Identifiability is motivating because a group member expects a positive outcome due to her own, individual contribution (Karau and Williams, 1993). Such positive outcomes include earning praise, receiving an individual reward for exceptional performance, or outperforming the other group members. Because these positive outcomes all apply to a single group member and not to the entire group, the goal matching this mechanism is best described as individual (e.g., “I want to win”; Kerr et al., 2007). Thus, although individual goals are not necessarily competitive (cf. Van Lange, 1999; Murphy and Ackermann, 2014), they do not focus on the group outcome. In sum, then, collective as well as individual goals may motivate group members to perform well. In the present paper, we go one step further and analyze how group members act to attain these collective and individual goals.

### Goal Setting and Goal Striving: Setting Goals and Making Plans

The psychology of action (Gollwitzer and Bargh, 1996; Gollwitzer and Moskowitz, 1996) distinguishes between a first step of committing strongly to one’s goal (goal setting) and a second step where one has to implement goal-directed actions and responses—a process referred to as *goal striving* (Lewin, 1926; Lewin et al., 1944; Heckhausen and Gollwitzer, 1987; Gollwitzer, 1990). In support of the distinction between goal setting and goal striving, a meta-analysis (Webb and Sheeran, 2006) on experimental studies that manipulated the strength of the goal showed that a medium-to-large (Cohen, 1992) increase in commitment (*d* = 0.66) only led to a small-to-medium change in respective behavior (*d* = 0.36); an effect largely due to people who are strongly committed but fail to act on their intention (Sheeran, 2002). Fully understanding intention-behavior relations thus requires analyzing goal setting as well as goal striving.

What may be the actions and responses that group members choose to attain collective and individual goals during goal striving? Research on conflict resolution shows that positively interdependent goals lead to cooperative interaction, such as helping and talking to each other (Deutsch, 1949). In the context of conjunctive physical persistence, such cooperative interaction should surface in increased and more group focused verbal interaction, including encouragement, discussing the common goal, and how the group is doing (Deutsch, 2011). Given that collective goals highlight positive interdependence, group members should strive for them cooperatively exhibiting just this type of cooperative verbal interaction. Assuming that individual goals do not highlight positive interdependence, group members should strive for them less cooperatively. Testing this prediction thus not only requires setting collective goals (as commonly triggered by indispensability) versus individual goals (as commonly triggered by identifiability) but also supporting the respective goal striving route.

A simple way to support efficient goal striving is planning out in advance when, where, and how to strive for a set goal in an if-then format (e.g., “And if I encounter situation S, then I will show the goal-directed response R!”; Gollwitzer, 1993, 1999, 2014). By forming such IIs, one commits to performing the specified behavior in the pre-planned situation. Thereby, one is more likely to act on and attain one’s goal (Gollwitzer and Sheeran, 2006; Adriaanse et al., 2011; Bélanger-Gravel et al., 2013; Toli et al., 2016). In the present paper, we suggest that different types of IIs support striving for collective versus individual goals.

### Supporting Group Goal Striving: Forming Collective versus Individual Implementation Intentions

Recent research shows that if-then planning also increases the rate of goal attainment in small group performance (Wieber et al., 2012, 2013; Thürmer et al., 2015a). For instance, if-then planning has been shown to help groups attain their goal of making informed decisions in hidden profile situations (Thürmer et al., 2015b), to curb their investments in an escalation of commitment paradigm (Wieber et al., 2015), and to improve their performance in an interactive puzzle task (Wieber et al., 2017, unpublished). In some of these studies, groups used traditional IIs that refer to the individual (Wieber et al., 2017, unpublished); but in other studies, groups used new cIIs that refer to the group (Thürmer et al., 2015b; Wieber et al., 2015). Since all these studies consistently report that groups were more likely to attain their performance goal when forming additional if-then plans, group members seem to be able to use new cIIs as well as the traditional individual IIs to increase their performance. However, these reported studies did not compare the effects of cIIs and IIs in a single design, and a systematic test comparing the effects of individual versus cIIs as well as their underlying processes is still lacking.

We thus propose that group members can effectively regulate their goal striving by forming IIs that refer to the group (we, us, our). Like individual IIs, such cIIs are if-then plans that specify when, where, and how to act toward a set goal. Different from IIs that refer the individual (I, me, mine) and support an individual goal, cIIs refer to the group (e.g., “And if *we* encounter situation S, then *we* will show response R!”) and support a collective goal. If increases in cooperation are indeed due to effective goal striving (and not merely goal setting), supporting respective goal striving should magnify the differences between collective and individual goals. cIIs should activate the collective goal striving route and thus increase cooperation. Because IIs support the goals they are set for, referring to the group should therefore support cooperation. In contrast, an individual II may not activate cooperative strategies because it only refers to oneself. In sum, cIIs as well as IIs should increase group performance but cIIs should increase cooperation within the group.

### The Present Research

We analyzed whether planning out how to strive for collective and individual goals with respective cIIs and individual IIs increases performance and leads to more versus less cooperative interaction. We used a conjunctive physical persistence task where freely interacting groups were asked to hold a medicine ball as long as possible (Bray, 2004). We chose this task because each group member has to contribute equally and is therefore indispensable for performance; moreover, because group members are interacting face to face, it is easy to identify who failed first (Kerr et al., 2007; Weber and Hertel, 2007; Kerr and Hertel, 2011). According to our earlier reasoning that indispensability triggers collective goals and that identifiability triggers individual goals, supporting individual as well as collective goal striving with respective if-then plans should therefore improve performance. However, while cIIs should rely on cooperation to improve performance, this does not have to be the case for IIs.

In two experiments, groups either set individual or collective goals and then performed two rounds of the persistence task (baseline and experimental). After the baseline round, groups furnished their goal with a respective plan to ignore muscle pain and tell themselves they can do it (i.e., a cII or an individual II). Such if-then plans to ignore negative affect and to increase self-efficacy feelings have been found to be highly effective (e.g., Bayer and Gollwitzer, 2007; Schweiger Gallo et al., 2009; Thürmer et al., 2013). In Experiment 1, we further established two control conditions without respective if-then plans to assess whether forming additional if-then plans improves performance. We expected that both types of plans (i.e., cIIs and IIs) increase performance but lead to more versus less cooperative verbal interaction during task performance. In Experiment 2, we followed up on this assumed process and manipulated whether the task allowed for communication or hindered communication. Because we assumed that cIIs but not IIs rely on cooperative interaction to increase performance, we expected cIIs to lead to better performance when the task supported communication. IIs, on the other hand, should be effective when communication is hindered.

## Experiment 1: Do Collective Implementation Intentions Support Cooperative Interaction and Performance?

The aims of Experiment 1 were twofold: First, we sought to establish that cIIs and individual IIs improve group performance in comparison to respective goals. Because indispensability (which should usually trigger collective goals) and identifiability (which should usually trigger individual goals) improve performance in such tasks, we expected that cIIs as well as individual IIs improve performance in comparison to the respective mere goals. Second, we sought to investigate if cIIs indeed lead to different interaction patterns during goal striving than IIs. Positively interdependent goals commonly are associated with cooperative interaction, and supporting collective goals with cIIs should therefore intensify group interaction. In freely interacting groups, this intense group interaction should express itself in a high amount of verbal communication between group members. Related to this, the content of the verbal communication between group members should also reflect the respective type of goal striving. Research in psycholinguistics emphasizes that personal pronouns can be markers of one’s identity (Pennebaker et al., 2003) and research on social identity has shown that group-related pronouns can be indicative of group processes such as cooperation (Perdue et al., 1990; Brewer and Gardner, 1996). If cIIs support cooperative goal striving, group members with cIIs should therefore refer more to the group in their verbal communications by using first person plural pronouns (we, us, our) and use more cooperative words.

### Method

#### Participants and Design

One hundred and fifty-six students from the University of Konstanz (117 females) with a mean age of 22.58 years (*SD* = 4.40) participated in return for coffee vouchers, 4€ (i.e., about 5$), or partial course credit. Participants were invited to the laboratory in same-sex triads (52 triads, 39 female) and a male experimenter randomly assigned groups to a 2 (Implementation Intention: yes vs. no) × 2 (Referent: individual vs. collective) factorial design. We used triads instead of dyads because some group researchers have argued that group phenomena might operate differently or not even occur in dyads (see Moreland, 2010; Williams, 2010, for a discussion).

One participant in a cII group reported pain from a past injury (the trial was aborted immediately), one collective control group was not recorded because of hardware failure, two groups had members who were much older (3 SDs over the mean age of the sample; 1 collective control group, 1 II group), and one group stated during debriefing that they had formed IIs although they were in the collective control condition (including these groups in the analysis did not change the pattern of results); 47 triads (35 female) remained for analyses. A power analysis (1 – β = 0.70) with G^∗^Power (Faul et al., 2007, 2009) showed that our sample size allows detecting a medium-to-large effect ($\eta_{p}^{2}$ = 0.15) in our four-cell design.

#### Procedure

After obtaining informed consent including a general fitness check, the experimenter explained that the study investigated persistence in teams. Participants were to hold a ball simultaneously as long as possible by standing in a triangle and stretching out their dominant arm (Bray, 2004). For this task, groups were asked to form the goal “We (I) want to hold the ball as long as possible” (individual phrasing in parentheses) that was written on a board. Participants then performed the first round of the task and the experimenter measured how long the group persisted.

Next, participants received a paper-and-pencil form that included the manipulation of the referent and implementation intention factors. To test whether IIs improve persistence, experimental groups either added the collective if-then plan (cII) “And if our muscles hurt, then we will ignore the pain and tell ourselves: We can do it!” to their collective goal or the individual if-then plan (II) “And if my muscles hurt, then I will ignore the pain and tell myself: I can do it!” to their individual goal. The referent factor was thus manipulated by either referring to the group (we/collective) or to the individual (I). To make sure that individual and collective control groups had the same task-relevant knowledge, they were asked to add: “We (I) will ignore our (my) muscle pain and tell ourselves (myself): We (I) can do it!” (individual phrasing in parentheses). The content of these instructions therefore did not differ between conditions apart from the if-then structure of the IIs. Participants read the instructions individually, repeated their plans silently, envisioned them in their mind’s eye, and finally wrote them down. This procedure took about 5 min.

To measure the impact of this manipulation, a second, experimental round of the persistence task followed. To rule out the possibility that cIIs increase persistence because of increased goal commitment (a goal-setting variable), participants then responded to three goal commitment items (“It’s hard to take this goal seriously [reverse scored]/I am strongly committed to pursuing this goal/It wouldn’t take much to make me abandon this goal [reverse scored]” 1: *not agree at all* to 7: *agree completely*, *Cronbach’s* α = 0.71, *ICC*(1) = -0.12, *ICC*(2) = -0.47),^2^ adapted from Klein et al. (2001). Moreover, at the group level, increased group identification might improve performance, which also does not qualify as a goal striving process. Participants thus responded to seven group identification items (“I identify with my group/It is important to me to belong to my group/The fact that I belong to my group has little to do with how I see myself [reverse scored]/I am happy that I belong to my group/I often regret that I belong to my group [reverse scored]/I feel strong ties with my group/In general, I like belonging to my group” 1: *disagree* to 7: *agree completely*, *Cronbach’s* α = 0.84, *ICC*(1) = 0.26, *ICC*(2) = 0.51), adapted from Leach et al. (2008). Finally, participants provided demographic information including their height, were debriefed, thanked, and paid.

#### Dependent Measures

We recorded how long groups held the medicine ball in seconds per trial. As common in research on group persistence (e.g., Kerr et al., 2012), the difference between the experimental and the baseline measure was computed to measure the impact of the planning manipulation on persistence. The audio recordings made during the trials were transcribed by a research assistant, and the word count function of the computer program AtlasTi (Muhr, 2012) counted the number of words per trial. We used the difference between the baseline and the experimental round to measure the impact of the planning manipulation on verbal communication. Two independent coders identified words representing group cooperation (e.g., teamwork, support, help; inter-coder agreement = 72%). In line with linguistic research on pronouns and identity (Pennebaker et al., 2003), we further coded the first person plural pronouns (we, us, ours) in the cooperation category.

### Results and Discussion

Unless indicated otherwise, we analyzed the data with an ANOVA with Implementation Intention (yes vs. no) and Referent (individual vs. collective) as between factors.

#### Equivalence of Conditions and Baseline Analysis

All participants copied their respective goals and plans to the form correctly. Group identification scores, *M* = 5.26, *SD* = 0.96, and goal commitment scores, *M* = 5.99, *SD* = 0.78, were generally high and did not differ between conditions, independent of whether the scores were aggregated across group members or treated as independent, *F*s < 1, *p*s > 0.50. Increased motivation or stronger group identification therefore do not qualify as alternative explanations for the expected performance improvements through if-then planning.

Entering baseline persistence in a preliminary ANOVA surprisingly showed a marginal Implementation Intention × Referent interaction, *F*(1,43) = 3.87, *p* = 0.06, $\eta_{p}^{2}$ = 0.08. As the implementation intention factor was not manipulated until after this baseline measure, it is not plausible that the plan condition could have influenced persistence at that point. Instead, this effect may reflect different ability-levels of the groups. In line with this reasoning, the interaction became non-significant when entering the mean height of the group members as a covariate, *F*(1,42) = 1.83, *p* = 0.18, $\eta_{p}^{2}$ = 0.04. Accordingly, we calculated the difference between experimental and baseline measures (Round 2 – Round 1) to assess the impact of the manipulation; including height as a covariate did not change the following persistence analysis. No main or interaction effects for the number of words spoken, *F*s(1,43) < 1.90, *p*s > 0.17, or the number of cooperative words spoken, *F*s(1,43) < 2.90, *p*s > 0.10, were observed at baseline, and we therefore also calculated difference scores.

#### Dependent Variable: Persistence

**FIGURE 1:**
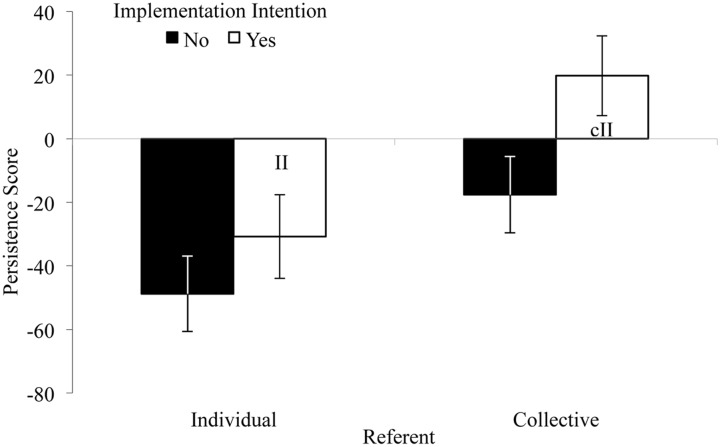
**Mean persistence scores (experimental [s] minus baseline [s]) by Implementation Intention and Referent (Experiment 1).** Error bars represent standard errors. II: Individual implementation intention; cII: collective implementation intention.

In line with previous research (e.g., Lount et al., 2008), groups deteriorated from baseline to experimental round (**Table 1**). To test whether forming if-then plans improved persistence, we entered the persistence score (experimental minus baseline) into the ANOVA. As expected, groups with an implementation intention (cII or II) persisted relatively longer in the experimental round than groups with a control plan, *F*(1,43) = 5.11, *p* = 0.03, $\eta_{p}^{2}$ = 0.11 (**Figure 1**). This supports our prediction that an II as well as a cII to ignore muscle pain and to tell oneself that one can do well on the task improves persistence. Moreover, a main effect of referent occurred, *F*(1,43) = 11.16, *p* < 0.01, $\eta_{p}^{2}$ = 0.21: Groups that had made collective plans persisted relatively longer than groups with individual plans. The main effects were not qualified by an Implementation Intention × Referent interaction, *F*(1,43) = 0.63, *p* = 0.43.^3^ In sum, the observed results are in line with the idea that if-then planning supports group performance. But do the two types of implementation intentions (cIIs and IIs) rely on different processes?

**Table 1:** Persistence, verbal communication, and communication content measures by Implementation Intention (II) and Referent (Experiment 1).

	Referent			
	
	Individual		Collective	
		
Measure	II: no	II: yes	cII: no	cII: yes
**Seconds holding the ball**	**Persistence**			
	
Baseline	190.86 (54.14)	212.60 (81.33)	193.58 (77.49)	139.82 (44.54)
	[159.60; 222.11]	[154.42; 270.78]	[144.35; 242.82]	[109.89; 169.74]
Experimental	142.07 (44.20)	181.80 (61.09)	176.00 (73.39)	159.64 (41.58)
	[116.55; 167.59]	[138.10; 225.50]	[129.37; 222.63]	[131.70; 187.57]
Difference (dependent measure)	-48.79 (28.75)	-30.80 (62.03)	-17.58 (39.53)	19.82 (34.76)
	[-65.39; -32.19]	[-75.17; 13.57]	[-42.70; 7.53]	[-3.54; 43.17]

**Number of words spoken**	**Verbal communication**			

Baseline	96.57 (86.62)	174.40 (135.78)	104.08 (94.44)	100.73 (84.81)
	[46.56; 146.59]	[77.27; 271.53]	[44.08; 164.08]	[43.75; 157.70]
Experimental	52.14 (60.81)	71.80 (104.68)	72.75 (76.82)	99.00 (91.02)
	[17.03; 87.25]	[-3.08; 146.68]	[23.94; 121.56]	[37.85; 160.15]
Difference (dependent measure)	-44.43 (56.94)	-102.60 (93.28)	-31.33 (67.93)	-1.73 (42.69)
	[-77.30; -11.56]	[-169.33; -35.87]	[-74.49; 11.83]	[-30.40; 26.95]

**Number of cooperative words spoken**	**Communication content**			

Baseline	3.21 (2.86)	5.40 (4.84)	3.75 (3.44)	2.64 (1.80)
	[1.56; 4.87]	[1.94; 8.86]	[1.56; 5.94]	[1.42; 3.85]
Pronouns	2.86 (2.51)	4.70 (4.40)	3.42 (3.40)	1.91 (1.38)
	[1.41; 4.30]	[1.55; 7.85]	[1.26; 5.57]	[0.99; 2.83]
Other	0.36 (0.84)	0.70 (0.90)	0.33 (0.65)	0.73 (0.90)
	[-0.13; 0.84]	[0.02; 1.38]	[-0.08; 0.75]	[0.12; 1.33]
Experimental	1.07 (1.86)	2.10 (4.33)	2.58 (2.87)	4.00 (4.75)
	[0.00; 2.14]	[-1.00; 5.20]	[0.76; 4.41]	[0.81; 7.19]
Pronouns	1.07 (1.86)	1.90 (4.01)	2.50 (2.84)	3.73 (4.45)
	[0.00; 2.14]	[-0.97; 4.77]	[0.69; 4.31]	[0.74; 6.72]
Other	0.00 (n/a)	0.20 (0.42)	0.08 (0.29)	0.27 (0.47)
	[n/a]	[-0.10; 0.50]	[-0.10; 0.27]	[-0.04; 0.59]
Combined difference (dependent measure)	-2.14 (2.28)	-3.30 (4.50)	-1.17 (2.48)	1.36 (3.80)
	[-3.46; -0.83]	[-6.52; -0.08]	[-2.74; 0.41]	[-1.19; 3.92]
*n*	14 triads	10 triads	12 triads	11 triads


*Standard deviations are in parentheses and 95% confidence intervals of the condition mean are in brackets.*

#### Process Measure: Group Interaction

We argued that cIIs support the use of cooperative task strategies in comparison to IIs. We thus expected that collective if-then plans would lead to more communication than individual if-then plans. To test this prediction, we entered the word count difference score (experimental minus baseline) into the ANOVA. Indeed, groups in the collective conditions spoke more to each other in the experimental round than groups in the individual conditions (**Table 1**), *F*(1,43) = 8.53, *p* = 0.01, $\eta_{p}^{2}$ = 0.17. However, this main effect was qualified by an Implementation Intention × Referent interaction, *F*(1,43) = 5.06, *p* = 0.03, $\eta_{p}^{2}$ = 0.11. Groups in the control conditions did not differ in the amount they spoke, *F*(1,43) = 0.25, *p* = 0.62, but the cII lead to more communication in comparison to the II, *F*(1,43) = 12.10, *p* < 0.01, $\eta_{p}^{2}$ = 0.22. Planned contrasts showed that the cII led to more communication than all other conditions, *t*(43) = 2.52, *p* = 0.02, and the II actually led to less communication than all other conditions, *t*(43) = 3.42, *p* < 0.01. Collective planning with the cII thus indeed lead to more verbal group interaction.

We also assumed that groups with cIIs should communicate more cooperatively than groups with IIs. To test this assumption, we entered the cooperation score into the ANOVA. This ANOVA showed a Referent main effect, *F*(1,43) = 7.41, *p* = 0.01, $\eta_{p}^{2}$ = 0.15, that was qualified by a marginal Referent × Implementation Intention interaction, *F*(1,43) = 3.66, *p* = 0.06, $\eta_{p}^{2}$ = 0.08. Pairwise comparisons showed that groups with cIIs spoke more cooperatively with each other than groups with IIs, *F*(1,43) = 10.60, *p* < 0.01, $\eta_{p}^{2}$ = 0.20; no Referent effect occurred for control groups, *F*(1,43) = 0.63, *p* = 0.43. One may argue that this effect is mainly driven by priming the collective referent “we;” however, even within the collective referent conditions that both referred to “we,” if-then planning with cIIs tended to increase cooperative communication, *F*(1,43) = 3.42, *p* = 0.07, $\eta_{p}^{2}$ = 0.07. This overall pattern of results is thus in line with our assumption that cIIs but not IIs support cooperative collective goal striving.

In sum, Experiment 1 shows that if-then planning improves group performance, and that cIIs and IIs lead to different group interaction patterns. While cIIs left verbal communication between group members intact and led group members to speak cooperatively with each other, IIs lead to less verbal communication. This pattern supports the assumption that group members can strive for goals collectively or individually, and that forming respective if-then plans supports the matching type of goal striving (collective or individual). We conducted Experiment 2 to confirm the causal impact of this assumed process by either supporting or hindering cooperative verbal interaction.

## Experiment 2: Do Collective Implementation Intentions Cause Performance Improvements Because of Group Interaction?

The aim of Experiment 2 was to directly test whether cIIs improve performance via cooperative group interaction. To test this process hypothesis, we opted to manipulate the assumed process variable (group interaction), as recommended by Spencer et al. (2005; see also Bullock et al., 2010). Our reasoning was as follows: If cIIs rely more on group interaction than IIs, hindering group interaction should impair performance in cII groups but not in II groups. To this end, groups were either encouraged to communicate with each other while performing the persistence task or were prevented from communicating. Moreover, groups formed either cIIs (collective referent) or IIs (individual referent) between the first and the second round. Assuming that cIIs achieved their effects by enhancing cooperative interaction between group members, we hypothesized that cIIs should lead to better performance when the task supported communication between group members but that this should not be true for IIs.

### Method

#### Participants and Design

One hundred and twenty-three university students (90 females) with a mean age of 22.13 years (*SD* = 2.85) participated in return for 4€ or partial course credit. Participants were invited to the laboratory in same-sex triads (41 triads, 30 females) and a female experimenter randomly assigned them to one of four conditions in a 2 (Implementation Intention Referent: individual vs. collective) × 2 (Communication: supported vs. hindered) between factorial design. Three groups (1 in the II condition and 2 in the cII condition) did not follow task instructions and communicated despite being prompted not to (their values were in the 95% CI range of the communication-supported condition); thirty-eight triads (27 females) remained for statistical analysis. A power analysis (1 - β = 0.70) with G^∗^Power (Faul et al., 2007, 2009) indicates that our sample size allows detecting an effect of the size observed in Experiment 1 ($\eta_{p}^{2}$ = 0.15) in our four-cell design.

#### Procedure

Experiment 2 followed the same procedure as Experiment 1, with the following difference: Before the first round of the task, instructions were varied to manipulate the Communication factor. To hinder communication, groups were instructed not to talk to each other, to each look at a separately marked point on the wall away from the group, and to wear a headset over their ears; to support communication, groups were told that they are allowed to talk to each other, that they should face each other, and wear the headset around their necks. Audio recordings were made as a manipulation check.

After forming the individual versus collective goals with IIs used in Experiment 1, groups performed the second round of the task. Participants then responded to the questionnaires assessing goal commitment [*Cronbach’s* α = 0.74, *ICC*(1) = -0.00, *ICC*(2) = -0.00]^2^ and group identification [*Cronbach’s* α = 0.91, *ICC*(1) = 0.13, *ICC*(2) = 0.32] used in Experiment 1. To check whether cII groups wanted to comply with their plan as much as II groups, participants also responded to a three-item questionnaire measuring plan commitment [“It is important for me to fulfill my plan/It would be a shame if I could not fulfill my plan/I feel committed to my plan,” 1: *not at all* to 5: *very much*, *Cronbach’s* α = 0.80, *ICC*(1) = 0.10, *ICC*(2) = 0.25] adapted from Wieber et al. (2015). Lastly, we asked participants for demographic information, including their major and semester of study.

### Results and Discussion

Unless indicated otherwise, we analyzed the data with an ANOVA with Implementation Intention Referent (collective: cII vs. individual: II) and Communication (supported vs. hindered) as between factors.

#### Equivalence of Conditions

All participants copied their respective goals and plans to the form correctly. Goal commitment, *M* = 5.10, *SD* = 0.78, plan commitment, *M* = 4.02, *SD* = 0.75, and group identification, *M* = 5.33, *SD* = 1.11, were high and did not differ between conditions, *F*s < 2.20, *p*s > 0.14. Participants across conditions thus equally wanted to comply with the adopted goals and plans, and cared about their group.

#### Persistence and Communication

To check whether the Communication factor manipulation was successful, we entered a word count of the first trial into the ANOVA: Groups in the communication-supported condition indeed spoke more than groups in the communication-hindered condition, *F*(1,34) = 25.67, *p* < 0.01, $\eta_{p}^{2}$ = 0.43 (**Table 2**). Even though groups in the “communication hindered” conditions did not manage to remain completely silent, the very large effect size ($\eta_{p}^{2}$ = 0.43 is equivalent to *d* = 1.68) suggests that our manipulation was successful. As expected, we neither observed an Implementation Intention Referent main effect, *F*(1,34) = 0.92, *p* = 0.34, $\eta_{p}^{2}$ = 0.03, nor an Implementation Intention Referent × Communication interaction, *F*(1,34) = 0.45, *p* = 0.51, $\eta_{p}^{2}$ = 0.01, at this point before the plan manipulation.

**Table 2:** Persistence measures by Implementation Intention Referent and Task Communication (Experiment 2).

	Implementation Intention Referent			
	
	Individual (II)		Collective (cII)	
		
Measure	Communication hindered	Communication supported	Communication hindered	Communication supported
	**Manipulation check: number of words spoken**			
	
Baseline	1.43 (3.37)	21.96 (12.33)	2.78 (5.69)	29.60 (24.62)
	[-0.98; 3.84]	[12.49; 31.44]	[-1.60; 7.15]	[11.99; 47.21]
Experimental	0.00 (n/a) [n/a]	12.78 (10.42)	0.00 (n/a) [n/a]	17.77 (13.07)
		[4.77; 20.79]		[8.42; 27.11]

	**Persistence**			
	
Baseline (s)	118.90 (43.24)	190.11 (60.43)	134.22 (57.63)	154.50 (44.17)
	[87.97; 149.83]	[143.66; 236.57]	[89.93; 178.52]	[122.90; 186.10]
Experimental (s)	120.00 (61.10)	139.22 (37.47)	97.22 (28.28)	132.20 (51.37)
	[76.29; 163.71]	[110.42; 168.02]	[75.49; 118.96]	[95.45; 168.95]
Difference	1.10 (42.37)	-50.89 (48.60)	-37.00 (34.97)	-22.30 (25.86)
	[-29.21; 31.41]	[-88.25; -13.53]	[-63.88; -10.12]	[-40.80; -3.80]
Difference *z*-transformed per communication condition (dependent measure)	1.84 (4.32)	-1.64 (5.30)	-2.05 (3.57)	1.48 (2.82)
	[-1.25; 4.93]	[-5.72; 2.43]	[-4.79; 0.70]	[-0.54; 3.49]
*n*	10 triads	9 triads	9 triads	10 triads


*Standard deviations are in parentheses and 95% confidence intervals of the condition mean are in brackets.*

To test whether the communication manipulation alone impacted performance, the first round persistence measure was entered into the ANOVA. Groups in the communication-supported condition outperformed groups in the communication-hindered condition (**Table 2**), *F*(1,34) = 7.48, *p* = 0.01, $\eta_{p}^{2}$ = 0.18. This suggests that intense interaction increases performance in our physical persistence task, which is consistent with the Referent main effect observed in Experiment 1. As expected, we neither observed an Implementation Intention Referent main effect, *F*(1,34) = 0.37, *p* = 0.55, $\eta_{p}^{2}$ = 0.01, nor an Implementation Intention Referent × Communication interaction, *F*(1,34) = 2.32, *p* = 0.14, $\eta_{p}^{2}$ = 0.06, at this point before the plan manipulation.

#### Persistence and If-then Planning

**FIGURE 2:**
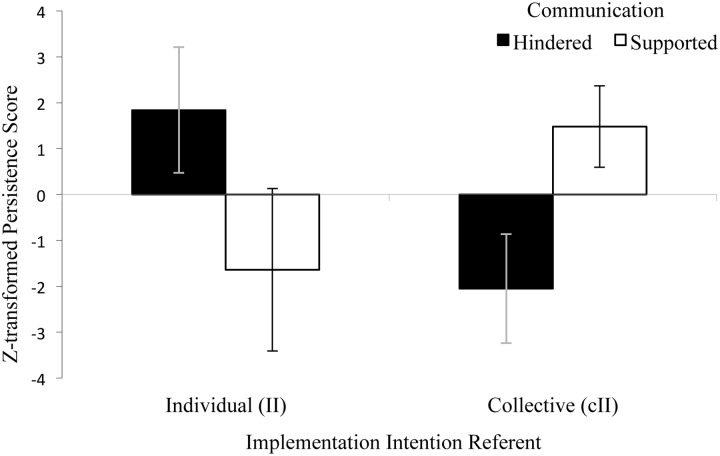
***z*-transformed persistence scores by Implementation Intention Referent and Communication (Experiment 2).** Error bars represent standard errors. II: Individual implementation intention, cII: collective implementation intention.

We next tested how collective and individual if-then planning impacted performance and thus again calculated the persistence score (experimental minus baseline). Because we were interested in the additional effects of planning, we sought to account for the systematic baseline differences caused by the Communication factor. We therefore pooled the persistence difference scores per Communication condition (i.e., collapsed across Implementation Intention Referent conditions) and then computed the respective *z*-scores (**Table 2**). Entering this score as dependent variable into the ANOVA revealed the expected Implementation Intention Referent × Communication interaction, *F*(1,34) = 6.98, *p* = 0.01, $\eta_{p}^{2}$ = 0.17: As predicted, cII groups marginally performed better when communication was supported (**Figure 2** and **Table 2**), *F*(1,34) = 3.53, *p* = 0.07, $\eta_{p}^{2}$ = 0.09.^4^ Thus, hindering communication impaired performance in cII groups.

Moreover, II groups marginally performed better when the communication was hindered, *F*(1,34) = 3.45, *p* = 0.07, $\eta_{p}^{2}$ = 0.09. This may suggest that communication without the focus on interdependence that collective goals and plans provide may be distracting instead of helpful. Future research should investigate this hypothesis.

#### If-then Planning and Communication

We also checked whether if-then planning changed verbal interaction in the communication condition. Entering the communication difference score (experimental minus baseline) into the model showed no main or interaction effects, all *F*s(1,34) < 2.76, all *p*s > 0.10; also no effects evinced when entering the cooperation score into the model, all *F*s(1,34) < 1, all *p*s > 0.65. Thus, cIIs did not further increase cooperative group interaction in Experiment 2. One reason for this finding may be that the groups who were instructed to communicate perceived the task as being highly cooperative and thus already interacted intensely during baseline. Future research should test this hypothesis.

Experiment 2 investigated whether group communication indeed qualifies as a process causing the positive effects of cIIs on group performance. We tested this assumption by manipulating whether the task at hand supported or hindered group communication. We found that cII effects were greater when communication was supported, whereas individual II effects were greater when communication was hindered. Together with Experiment 1 demonstrating that cIIs support cooperative verbal interaction, Experiment 2 suggests that cooperative group interaction does qualify as a process variable for cII effects but not II effects. Accordingly, it seems justified to distinguish between cIIs and individual IIs.

## General Discussion

Small group research has consistently identified two main motivators to work hard in a group: your group needs you (indispensability; Kerr and Hertel, 2011) and the other group members can recognize your contribution (identifiability; Karau and Williams, 1993). We argued that indispensability triggers collective goals (e.g., we want to beat our record), whereas identifiability triggers individual goals (e.g., I want to beat my record). The psychology of action highlights that, in addition to setting goals, people also need to effectively implement goal-directed actions to secure goal attainment. Accordingly, we argued that group members should strive for collective goals by cooperating with each other but strive for individual goals with less cooperation. Supporting striving for collective goals with cIIs should therefore enhance group performance via intensifying cooperative interaction.

Two experiments support this hypothesis and show that cIIs improve group performance in a conjunctive physical persistence task via cooperative verbal communication. The beneficial effects of cIIs on group performance rely on improved goal striving, as II effects on individual goal attainment: We observed performance improvements through cIIs when control groups received almost identical goals (same strategy with the same referent) only lacking the if-then format that is typical of IIs (Experiment 1). Moreover, the observed performance improvements by cIIs were not due to heightening participants’ goal commitment, but cIIs did increase cooperative group interaction. We manipulated the intensity of group interaction in Experiment 2 to confirm the causal role of this assumed process: Groups with cIIs but not groups with IIs performed worse when the task hindered communication. Apparently, enhanced cooperative interaction qualifies as a process associated with cII but not II effects.

### Implications for Small Group Performance

Observing that cIIs improve persistence via communication is in line with recent research showing that social support can lead to group motivation gains (Hüffmeier et al., 2014). But our research also demonstrates that groups may perform effectively without interacting (i.e., by striving individually), as is commonly found in the management literature (Lount and Wilk, 2014). Our research thus suggests that individual goals and plans may lead groups to perform well in conjunctive tasks, although not to the level of collective goals and plans.

It is important to note that group members’ personality attributes may moderate our effects. Implementation intention research shows that highly conscientious individuals do not benefit much from receiving additional implementation intention instructions, supposedly because they already plan spontaneously (Webb et al., 2007). Analogously, the conscientiousness of the average member (cf. Neuman et al., 1999) might moderate cII effects. Future research should test this assumption.

Our approach to motivation in groups may remind the reader of the work on goal setting and group performance (O’Leary-Kelly et al., 1994; Kleingeld et al., 2011). However, although forming IIs and setting challenging-and-specific goals both add specificity to one’s goal, these kinds of specificity differ: In goal setting, one quantifies the desired outcome (i.e., one specifies a certain goal standard), which makes discrepancies between the actual state and the desired end state easier to detect. In contrast, IIs specify how to attain an already set goal in terms of when, where, and how to act toward it. Despite these differences between IIs and goal setting, the individual-collective distinction is crucial for both: Collective goals (Locke and Latham, 1990; Crown and Rosse, 1995; Crown, 2007) as well as cIIs improve performance by improving group interaction. Groups can thus actively regulate the interaction between group members. This growing body of research is in line with the idea that groups are intentional entities that can regulate their behavior.

### Implications for the Psychology of Collective Action

Traditionally, the psychology of action and implementation intention research have focused on individuals (Gollwitzer and Sheeran, 2006; Gollwitzer and Oettingen, 2011), and research on implementation intention effects in groups is fairly recent (Wieber et al., 2012, 2013; Thürmer et al., 2015a). In the present research, we systematically investigated the implementation intention referent (i.e., We vs. I) and found that small groups can improve their performance by forming if-then plans that refer to the group (i.e., “we” if-then plans or cIIs). If-then planning is therefore not only effective in individuals and with an individual referent but also in groups and with a collective referent.

Recent accounts have discussed small group self-regulation without pointing to if-then planning. First, small group approaches address how groups attempt to regulate their members’ behavior (e.g., through assigning roles and enforcing norms) and how the group members react (e.g., by capitulating or resisting; Peterson and Behfar, 2005; Levine et al., 2010). Our perspective is complementary to this view: To use cIIs, group members may have to accept pertinent roles and norms because they otherwise lack commitment to collective goals. Second, the *group based self-regulation* account (Sassenberg and Woltin, 2008; Jonas et al., 2010; Woltin and Sassenberg, 2015) assumes that by identifying as a group member, one self-regulates in the service of a group. The self-regulation processes are assumed to be the same as those at the individual level. Intra-individual processes, such as committing to a goal and a plan, are also crucial for implementation intention effects. However, our distinction between cIIs and IIs is based on the referent (We vs. I) instead of the identification. In line with our perspective, cIIs increased cooperative communication but did not change group identification. A group member may thus identify with the group and still pursue goals individually. Lastly, others have also distinguished collective and individual motivation (e.g., Latham and Locke, 2007; Maciejovsky and Budescu, 2007, 2013; De Dreu et al., 2008; Levine and Smith, 2013; Maciejovsky et al., 2013). However, all these accounts have not distinguished between the incentive (goal) and the strategy to attain it (goal striving). In the light of modest intention-action relations (Sheeran, 2002; Webb and Sheeran, 2006), introducing this distinction is a crucial contribution of the present research.

In sum, the present research demonstrates that planning out collective and individual goals improves group performance via two different routes: Furnishing collective goals with cIIs increases cooperative interaction but furnishing individual goals with IIs does not. In this way, goal striving in groups with IIs helps groups perform to their full potential.

## Ethics Statement

This study was carried out in accordance with the recommendations of ethics committee of the University of Konstanz. All subjects gave written informed consent in accordance with the Declaration of Helsinki.

## Author Contributions

JLT, FW, and PG jointly designed the studies. JLT and FW supervised data collection and analyzed the data. JLT prepared a first draft and JLT, FW, and PG jointly revised the manuscript. This research is part of JLT’s doctoral dissertation.

## Conflict of Interest Statement

The authors declare that the research was conducted in the absence of any commercial or financial relationships that could be construed as a potential conflict of interest. The handling Editor declared a past co-authorship with one of the authors PG and states that the process nevertheless met the standards of a fair and objective review.

## Footnotes

1 Small group research has traditionally investigated indispensability and identifiability by manipulating task demands. In the present research, we focus on the individual’s own perspective that can emphasize one or the other process.

2 We used the formulas provided by Bliese (1998). Because of the poor agreement between group members, we additionally analyzed all scales assuming independence/without aggregating to the group level. This did not change the results. In Experiment 1, two items were excluded from the Klein et al. scale (“Quite frankly, I don’t care if I achieve this goal or not.” [reverse scored]/I think this is a good goal to shoot for”) because they reduced reliability (α < 0.50).

3 Entering gender as a covariate had a marginal main effect, *F*(1,42) = 3.86, *p* = 0.06, $\eta_{p}^{2}$ = 0.08, but it did not change the observed referent effect, *F*(1,42) = 9.44, *p* < 0.01, $\eta_{p}^{2}$ = 0.18, and the implementation intention effect, *F*(1,42) = 5.68, *p* = 0.02, $\eta_{p}^{2}$ = 0.12.

4 Entering gender as a covariate into the model did not have an effect, *F*(1,33) = 1.54, *p* = 0.22, and did not change the observed Implementation Intention Referent × Communication interaction, *F*(1,33) = 7.25, *p* = 0.01, $\eta_{p}^{2}$ = 0.18. As a proxy of familiarity, we coded how many group members had the same major and also were in the same semester (i.e., 1 = all group members are in a different semester or have a different major, 2 = two of the members are in the same semester and also have the same major, 3 = all group members have the same major and are in the same semester). Entering this variable as a covariate into the model neither had a main effect, *F*(1,33) = 0.43, *p* = 0.52, $\eta_{p}^{2}$ = 0.01, nor did it change the observed interaction, *F*(1,33) = 6.94, *p* = 0.01, $\eta_{p}^{2}$ = 0.17.
